# Intracochlear Perfusion of Tumor Necrosis Factor-Alpha Induces Sensorineural Hearing Loss and Synaptic Degeneration in Guinea Pigs

**DOI:** 10.3389/fneur.2019.01353

**Published:** 2020-02-10

**Authors:** Sachiyo Katsumi, Mehmet I. Sahin, Rebecca M. Lewis, Janani S. Iyer, Lukas D. Landegger, Konstantina M. Stankovic

**Affiliations:** ^1^Eaton Peabody Laboratories, Department of Otolaryngology—Head and Neck Surgery, Massachusetts Eye and Ear, Boston, MA, United States; ^2^Department of Otolaryngology—Head and Neck Surgery, Harvard Medical School, Boston, MA, United States; ^3^Program in Speech and Hearing Bioscience and Technology, Harvard Medical School, Boston, MA, United States; ^4^Harvard Program in Therapeutic Science, Harvard Medical School, Boston, MA, United States

**Keywords:** sensorineural hearing loss, cochlear synaptopathy, tumor necrosis factor-alpha, vestibular schwannoma, unpaired (orphaned) ribbons

## Abstract

Tumor necrosis factor-alpha (TNF-α) is a proinflammatory cytokine that plays a prominent role in the nervous system, mediating a range of physiologic and pathologic functions. In the auditory system, elevated levels of TNF-α have been implicated in several types of sensorineural hearing loss, including sensorineural hearing loss induced by vestibular schwannoma, a potentially fatal intracranial tumor that originates from the eighth cranial nerve; however, the mechanisms underlying the tumor's deleterious effects on hearing are not well-understood. Here, we investigated the effect of acute elevations of TNF-α in the inner ear on cochlear function and morphology by perfusing the cochlea with TNF-α *in vivo* in guinea pigs. TNF-α perfusion did not significantly change thresholds for compound action potential (CAP) responses, which reflect cochlear nerve activity, or distortion product otoacoustic emissions, which reflect outer hair cell integrity. However, intracochlear TNF-α perfusion reduced CAP amplitudes and increased the number of inner hair cell synapses without paired post-synaptic terminals, suggesting a pattern of synaptic degeneration that resembles that observed in primary cochlear neuropathy. Additionally, etanercept, a TNF-α blocker, protected against TNF-α-induced synaptopathy when administered systemically prior to intracochlear TNF-α perfusion. Findings motivate further investigation into the harmful effects of chronically elevated intracochlear levels of TNF-α, and the potential for etanercept to counter these effects.

## Introduction

Hearing loss is the most common sensory deficit in the world, affecting 466 million people today, and projected to affect 900 million by 2050 (1). The most common type of deafness is sensorineural hearing loss (SNHL), which is caused by damage to the delicate mechanosensory cells and auditory nerve fibers that reside within the inner ear's cochlea. The cochlea is a small, snail-shaped organ that receives acoustic information from the environment and relays it to the brain. SNHL remains largely irreversible, incurable, and poorly understood in humans due primarily to the cochlea's inaccessibility and the fact that its sensory epithelium, the organ of Corti, cannot be biopsied without damaging hearing.

Although SNHL is typically caused by aging, overexposure to noise or ototoxic drugs, or genetic mutation, an additional potentially life-threatening cause of SNHL is vestibular schwannoma (VS), a non-malignant tumor arising from Schwann cells of the vestibular portion of the eighth cranial nerve, and extending into the cerebellopontine angle of the brain. A striking 95% of VS patients develop SNHL; additional symptoms can include tinnitus, balance difficulties, and facial paralysis (2). The conventional hypothesis regarding the mechanism underlying VS-induced SNHL is that the tumor compresses the vestibulocochlear nerve, preventing neural signals carrying hearing and balance information from traveling from the inner ear to the brain; however, evidence from multi-site, large cohort studies suggests that associations between tumor size and location and SNHL severity are weak (3–7), and that SNHL may (4, 8, 9) or may not (8, 10–13) worsen with tumor growth over time in VS patients. Furthermore, (1) increased outer hair cell-generated distortion product otoacoustic emission (DPOAE) thresholds are observed in VS patients with minimal SNHL, indicating that outer hair cell damage may occur as a primary event, rather than secondary to neuronal damage (14), (2) 90% of human temporal bones from patients with ipsilateral VS show hair cell and neuronal damage that do not correlate with tumor size (15), and (3) biochemically-measured and radiologically-inferred intracochlear fluid protein levels are elevated in ears from poor-hearing VS tumor patients independent of tumor size (16–18).

These reports motivate the hypothesis that VS-induced SNHL is caused, at least in part, by VS-secreted factors that directly damage cochlear hair cells and neurons. Indeed, our laboratory has previously shown that human VSs grouped based on patient hearing ability tend to have different gene expression profiles, suggesting molecular contributions to VS-induced SNHL (19). Focusing specifically on VS secretions that could alter cochlear function by traveling through the internal auditory canal, our group previously identified a significant correlation between levels of VS-secreted pro-inflammatory cytokine tumor necrosis factor alpha (TNF-α) and the degree of a patient's SNHL (20). We further demonstrated that when VS secretions from poor hearing-patients are added to murine cochlear organotypic cultures, the typically resulting cellular damage can be partially prevented by neutralizing TNF-α in these secretions (20). These results strongly implicate TNF-α as a mediator of cochlear degeneration in response to VS secretion exposure, and motivate further investigation into the mechanisms by which TNF-α exerts its impact on cochlear structure and function.

TNF-α is a pro-inflammatory cytokine with key functions in diverse cellular processes, including regulation of the pro-inflammatory responses and maintenance of cellular homeostasis. While the expression of TNF-α is typically undetectable in the normal cochlea (21), it can be induced with aging (22), inflammation (23), noise trauma (24, 25), vibration (21), and exposure to ototoxic drugs including cisplatin, a platinum-based chemotherapeutic, and gentamicin, an aminoglycoside antibiotic (26) in animal models. In humans, elevated TNF-α serum levels have been reported in patients with idiopathic sudden sensorineural hearing loss (ISSNHL) (27) and autoimmune inner ear disease (AIED) (28).

Here, we investigate the effects of *in vivo* intracochlear TNF-α perfusion on cochlear structure and function in guinea pigs. These experiments have implications for understanding the mechanisms of VS-induced SNHL in addition to other forms of SNHL, including ISSNHL, AIED, and noise-induced hearing loss. We perfused TNF-α via the round window, which opens into the perilymph-filled scala tympani, because (a) VS-secreted TNF-α is likely to reach the cochlea via the fundus of the internal auditory canal, which is continuous with the cochlea's perilymph-filled lumina, (b) perilymph bathes the vast majority of cochlear cells, and (c) the round window is surgically accessible in a minimally invasive fashion, using an approach that is similar to the approach routinely used in humans for cochlear implantation. We show that acute *in vivo* TNF-α perfusion leads to an increase in the number of synaptic ribbons without paired post-synaptic receptors, suggesting synaptic degeneration. We also demonstrate the efficacy of etanercept, a TNF-α-inhibitor, for protecting against TNF-α-induced synaptic damage when administered prior to TNF-α. Results suggest a TNF-α-induced pathologic profile that is similar to what is observed in primary cochlear neuropathy.

## Materials and Methods

### Animals and Surgical Procedures

**Figure 1:**
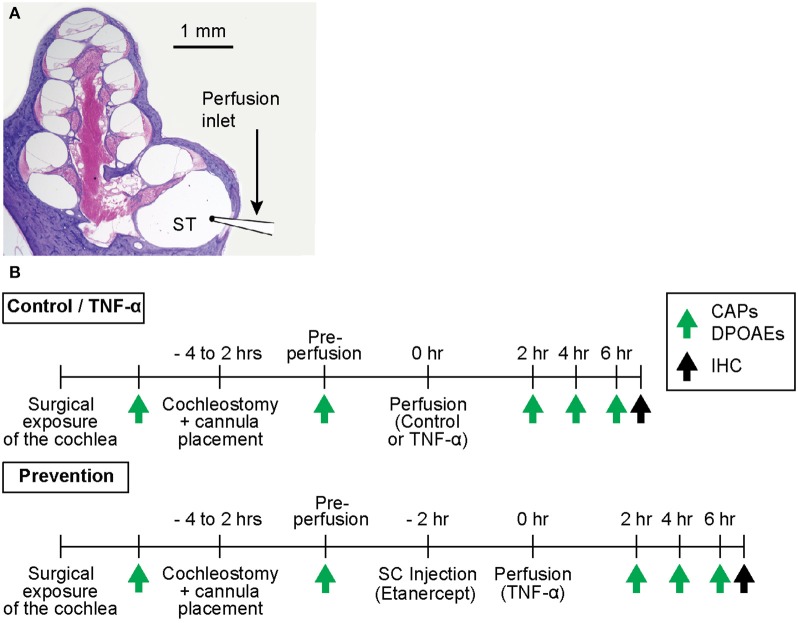
**(A)** Mid-modiolar cross-section through a hematoxylin and eosin (H&E)-stained guinea pig cochlea, depicting its four spiraling turns. The microcannula is positioned through a cochleostomy adjacent to the round window to enable slow cochlear perfusion through scala tympani (ST). **(B)** Experimental timelines for control and TNF-α experiments vs. prevention experiments. The timelines are identical aside from the subcutaneous (SC) injection of a TNF-α-blocker (etanercept) prior to perfusion of TNF-α in prevention experiments. Green arrows indicate time points of hearing tests (CAPs and DPOAEs), black arrows indicate time point of post mortem immunohistochemistry (IHC).

The experimental timeline is outlined in Figure 1. All procedures were approved by the Massachusetts Eye and Ear Institutional Animal Care and Use Committee.

Thirteen male albino guinea pigs (Hartley strain; 300–350 g; Charles River Laboratories, Inc., Wilmington, MA) were randomly assigned to one of three experimental groups: control (artificial perilymph, *n* = 5), TNF-α (TNF-α, *n* = 4), and prevention (etanercept + TNF-α, *n* = 4). Animals were anesthetized through intraperitoneal injections of pentobarbital sodium (Nembutal; 25 mg/kg), and intramuscular injections of fentanyl (0.2 mg/kg) and haloperidol (10 mg/kg) as described previously (29). Supplemental doses of 0.07 mg/kg Fentanyl and 3.0 mg/kg Droperidol alternating with 6.25 mg/kg Nembutal were administered as needed to maintain deep anesthesia (30). A single subcutaneous injection of Atropine (0.04 mg/kg) was provided to minimize salivation and prevent airway edema. Lidocaine (<15 mg/kg, 1 mL) was administered subcutaneously in the periauricular region and external auditory canal (avoiding the middle ear region) to minimize local pain.

A retroauricular incision was made to expose the tympanic bulla. A sharp blade was used to open the bulla and expose the basal turn of the cochlea and the round window. A silver wire electrode was positioned at the round window niche to record CAPs as described below, and a small surgical pick was used to place a cochleostomy 0.5 mm anterior to the round window. A perfusion microcannula was inserted into the cochleostomy (Figure 1A) and was fixed to the bulla with glue. A silicon globe stopper was positioned 3 mm from the cannula's tip to prevent the cannula from entering too far into scala tympani, and to seal the cochleostomy. The distal end of the microcannula was connected to a Hamilton syringe filled with control artificial perilymph (130 mM NaCl, 3.5 mM KCl, 1.5 mM CaCl_2_, 5.5 mM glucose, and 20 mM HEPES; at pH 7.5), or TNF-α (Recombinant Guinea Pig TNF-α Protein; 5035-TG-025 CF, R&D Systems, Inc., Minneapolis, MN; reconstituted to 100 μg/mL in phosphate buffered saline and diluted to 10 μg/mL in artificial perilymph) according to the condition being tested. The TNF-α concentration selected for the present experiments is higher than what we previously reported for human VS secretions (20) because we are using acute animal studies to model chronic and typically slowly-progressing VS-induced hearing loss in humans. Controlled perfusion was achieved using a pump (Harvard Apparatus PHD 2000, Harvard Apparatus, Holliston, MA) set to facilitate perfusion at a rate of 1.5 μL/min over the course of 50 min. For control and TNF-α experiments, a total volume of 7.5 μL of artificial perilymph or TNF-α, respectively, was perfused through scala tympani. For prevention experiments, 0.15 mL of etanercept (Enbrel, Amgen Inc., Thousand Oaks, CA) was injected subcutaneously 2 h prior to TNF-α perfusion (Figure 1B). Hearing was tested over the course of each experiment as described below. All experiments were carried out in an electrically- and acoustically-shielded, temperature-controlled chamber.

Animals were euthanized at the experiment's termination through intraperitoneal injections of Fatal Plus (Vortech Pharmaceuticals, Dearborn, MI), followed by intracardiac perfusion with 4% paraformaldehyde (PFA). Inner ears were immediately extracted and prepared for histological assessment as described below.

### Hearing Tests

Hearing was assessed via compound action potentials (CAPs), a measure of the synchronous firing of a population of auditory nerve fibers, and distortion product otoacoustic emissions, a proxy for outer hair cell function, over the course of each experiment. Specifically, hearing was tested just prior to cochleostomy placement, after positioning of the microcannula, and every 2 h after the first intracochlear perfusion until the experiment's termination (typically 6–8 h after the first perfusion).

All sound stimuli were delivered via a custom acoustic assembly comprising two dynamic drivers as sound sources and a miniature electret microphone to measure the sound pressure level (SPL) at the animal's ear (31, 32). Responses to auditory stimuli were monitored using a National Instruments PXI stimulus generation/data acquisition system (National Instruments, Austin, TX). The system was controlled and measurements were recorded using the Eaton Peabody Laboratories Cochlear Function Test Suite (33). All thresholds were measured and recorded at 8 frequencies from 2 to 32 kHz.

CAPs were recorded in response to tone-pip stimuli (0.5 ms duration, 0.5 ms rise-fall; cos^2^ onset envelope; 16/s) delivered at SPLs from 10 to 85 dB SPL proceeding in 5 dB steps. The electrode response was amplified (10,000X), filtered (0.3–3 kHz bandpass), and averaged (128 samples at each frequency-level combination). Threshold was defined as the lowest stimulus level at which a repeatable wave could be identified in the response waveform. The minimum input tone pip SPL that could generate a CAP amplitude of at least 0.4 μV was taken to be the CAP threshold of hearing; this value was empirically determined to be the voltage at which CAP amplitudes began to rise linearly with increasing SPL in our system. If the CAP amplitude was <0.4 μV at the maximum tone-pip SPL of 85 dB, the threshold was set to 85 dB.

When two primary tones f1 and f2 are simultaneously played to the inner ear, the cochlea generates acoustic distortion products that propagate back out through the inner and middle ear regions and can be recorded in the external auditory canal using a sensitive microphone. Distortion product otoacoustic emissions (DPOAEs) corresponding to the frequency 2f1–f2 are robust and are frequently measured to assess outer hair cell motility, which is required to generate the distortion products.

These DPOAEs were recorded as response amplitude vs. primary level functions (L1 = 10–80 dB; L2 = L1–10 dB; f1 and f2 incremented together in 5 dB steps); f2/f1 = 1.2 in the ear canal was amplified and digitally sampled at a rate of 200 kHz. Fourier analysis was used to determine the magnitudes of the DPOAE responses at f1, f2, and 2f1–f2. In a DPOAE magnitude spectrum, for a given frequency f, the noise floor was calculated as the average of the response magnitudes at the two frequencies above f and the two frequencies below f. The minimum input SPL required to generate a 2f1–f2 response with magnitude greater than the noise floor at 2f1–f2 was taken to be the DPOAE threshold.

### Histology

Immediately following euthanasia and intracardiac perfusion with 4% PFA, inner ears were extracted and dissected to remove extraneous bulla and tissue. Both the round and oval windows were opened and PFA was perfused through the scala tympani and scala vestibuli to ensure thorough fixation of the organ of Corti. Cochleae remained in 4% PFA for 2 h on a shaker and were subsequently placed in 0.12 M EDTA for 2 weeks to decalcify the otic capsule for whole mount preparation.

Histologic sections of the organ of Corti were prepared as previously described (34). In brief, following decalcification, the otic capsule was removed from the cochlea and the spiraling cochlea was microdissected into 10 pieces. Each piece was further microdissected to fully expose the organ of Corti; the spiral ligament, stria vascularis, and tectorial and Reissner's membranes were removed. The resulting cochlear whole mount sections were blocked in 5% normal horse serum (NHS; Sigma-Aldrich, St. Louis, MO) in 1% Triton X-100 (Integra Chemical, Kent, WA) for 1 h, followed by overnight incubation with primary antibodies against hair cell cytoplasm and stereocilia (MYOVIIa rabbit anti-Myosin VIIa from Thermo Fisher Scientific at 1:100 dilution), neurofilament (NF-H, chicken anti-Neurofilament heavy chain from Millipore Sigma, Burlington, MA at 1:200), a synaptic ribbon protein (CtBP2, mouse IgG1 anti-C-terminal binding domain protein 2 from BD Transduction Laboratories at 1:200), and a post-synaptic receptor protein (GluA2, mouse IgG2a anti-Glutamate Receptor A2, from Millipore at 1:100). Following overnight primary incubation at 30°C, each piece was rinsed in PBS three times, and then incubated in secondary antibody diluted in 1% NHS with 1% Triton-X 100. Secondary antibodies included goat anti-rabbit Pacific Blue, goat anti-chicken Alexa 488, goat anti-mouse IgG1 Alexa 568, and goat anti-mouse IgG2a Alexa 647 (all secondaries from Thermo Fisher Scientific at 1:200). Cochlear whole mount pieces were incubated in secondary antibody for two 1-h sessions, and then rinsed in PBS three times. Stained tissue was mounted under coverslips on glass slides with Vectashield mounting media (Vector Laboratories, CA, #H1000).

### Imaging and Synapse Quantification

Whole mounts were imaged using a Leica TCS SP8 laser-scanning confocal microscope. Preliminary low magnification (10X) images were acquired and subsequently arranged from base to apex and labeled according to frequency region (5.6, 8, 16, 24, and 32 kHz) using a custom ImageJ plug-in (35). In each dissected piece of the cochlea, a series of arcs is traced over the heads of the pillar cells with this plug-in to compute relative distances along the spiral. These distances are converted into best frequency based on a published cochlear frequency map for the guinea pig (36). The generated frequency maps guided subsequent high magnification (63X) imaging to visualize pre-and post-synaptic processes in the basal region of each inner hair cell.

High magnification z-stacks were uploaded to Amira 3D Software for Life Sciences (Thermo Fisher Scientific) for automated identification and segmentation of ribbons. Specifically, custom software (35) was used to locate each CtBP2-representative punctum and display the voxel space immediately surrounding it as a separate image, facilitating clear visualization of paired (with post-synaptic GluA2 receptor) vs. unpaired (orphaned) ribbons. Paired and orphaned ribbons were then counted manually for each frequency region and for each experimental condition.

### Statistical Analysis

**Figure 2:**
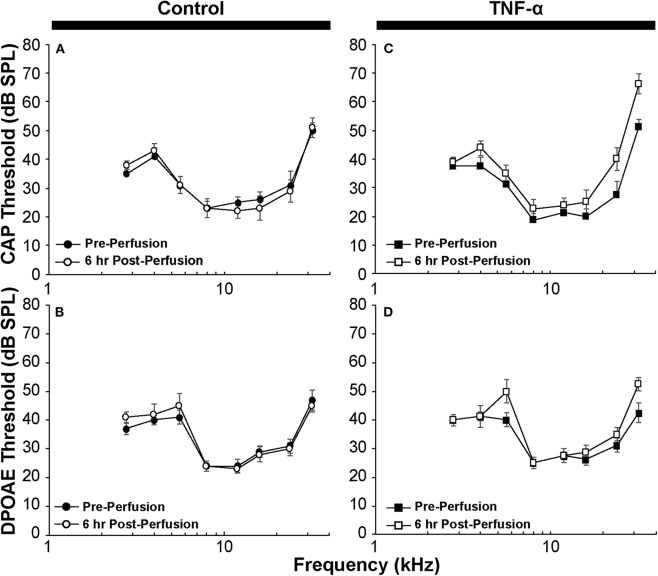
**(A)** CAP thresholds and **(B)** DPOAE thresholds in control guinea pig before vs. 6 h after perfusion of artificial perilymph through scala tympani demonstrate minimal impact of the surgical and perfusion techniques; *N* = 5 animals. **(C)** CAP thresholds and **(D)** DPOAE thresholds in the TNF-α group before vs. 6 h after intracochlear perfusion of TNF-α; *N* = 4 animals. Error bars represent standard errors of the mean. Differences in thresholds were not statistically significant (*p* > 0.05).

Differences in DPOAE and CAP thresholds before and after intracochlear perfusion were assessed by two-way ANOVA. Error bars in Figure 2 indicate standard errors of the mean. When comparing differences in CAP amplitudes and the number of orphaned ribbons per inner hair cell among the three groups, Kruskal Wallis *H*-tests were applied using IBM SPSS and Microsoft Excel software.

## Results

**Figure 3:**
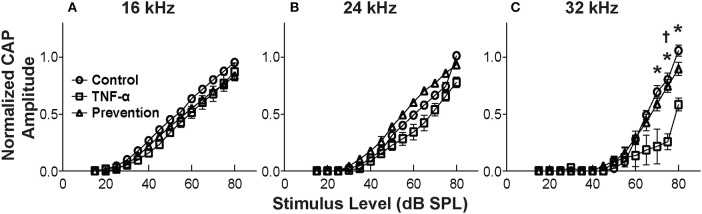
Mean CAP amplitude vs. level functions for 16 kHz **(A)**, 24 kHz **(B)**, and 32 kHz **(C)** for the control group (perfused with artificial perilymph), the TNF-α group and the prevention group (animals received etanercept 2 h prior to receiving TNF-α). Amplitudes are normalized to the 80 dB SPL pre-intracochlear perfusion response for each animal. Normalized amplitudes in TNF-α group are significantly decreased relative to control and prevention group at 32 kHz. ^*^, ^†^ = statistically significant at *p* < 0.05 for control vs. TNF-α and TNF-α vs. prevention groups, respectively. Error bars represent SEM. Figure legend in **(A)** also applies to **(B,C)**.

CAP thresholds (Figure 2A) and DPOAE thresholds (Figure 2B) did not change in response to intracochlear perfusion with artificial perilymph, indicating a consistent technique. While CAP (Figure 2C) and DPOAE (Figure 2D) thresholds 6 h post-TNF-α perfusion did not significantly change in the TNF-α group relative to control, and the growth of DPOAE as a function of sound level was not significantly affected among the groups (Supplemental Figure 1), there was a trend indicating an increase in CAP thresholds pre- vs. 6 h post-perfusion at 24 and 32 kHz in the TNF-α group (Figure 2C). This trend motivated subsequent analysis of physiologic and morphologic changes in these frequency regions. Specifically, we analyzed CAP amplitudes and afferent synapses between inner hair cells and auditory nerve fibers because previous research has shown that (a) CAP amplitude is a more sensitive metric of neuronal damage than CAP threshold (37), (b) TNF-α-mediated sensorineural damage localizes to the basal turn (38, 39), and (c) these synapses may be damaged even when audiometric thresholds are normal or near-normal (40, 41). For each animal, CAP amplitudes were normalized to the 80 dB SPL pre-intracochlear perfusion response (Figure 3). At 32 kHz, there were statistically significant differences in CAP amplitudes across the three experimental groups at multiple sound levels between 70 and 80 dB SPL. Specifically, the analysis at 80 dB SPL revealed χ^2^ (2) = 7.517, *p* = 0.01. Further analysis revealed a significant difference between the TNF-α and control groups [χ^2^ (1) = 6.05, *p* = 0.01], and a similar trend between the TNF-α and prevention groups [χ^2^ (1) = 4.133, *p* = 0.05], but importantly, not between the control and prevention groups [χ^2^ (1) = 0.96, *p* = 0.41]. Similarly, at 75 dB SPL, there was a significant difference in the normalized CAP amplitudes across the three groups [χ^2^ (2) = 7.736, *p* = 0.01]. Further, CAP amplitude was significantly reduced in the TNF-α group relative to the control group [χ^2^ (1) = 6.05, *p* = 0.01] and prevention group [χ^2^ (1) = 5.398, *p* = 0.02]; there was no difference between the control and prevention groups [χ^2^ (1) = 0, *p* = 1]. Finally, at 70 dB SPL, there was statistically significant difference across the three experimental groups [χ^2^ (2) = 9.972, *p* = 0.01]. Additional analysis revealed a significant difference between the TNF-α and control groups [χ^2^ (1) = 6.05, *p* = 0.01], and a similar trend between the TNF-α and prevention groups [χ^2^ (1) = 4.133, *p* = 0.05], but not between the control and prevention groups [χ^2^ (1) = 0.06, *p* = 0.90]. The reduced CAP amplitudes in the TNF-α group compared to the control group indicate a neurotoxic effect of TNF-α. In addition, the observed difference between the control and prevention groups is minimal, demonstrating the efficacy of etanercept for protecting against TNF-α-induced neurotoxicity.

**Figure 4:**
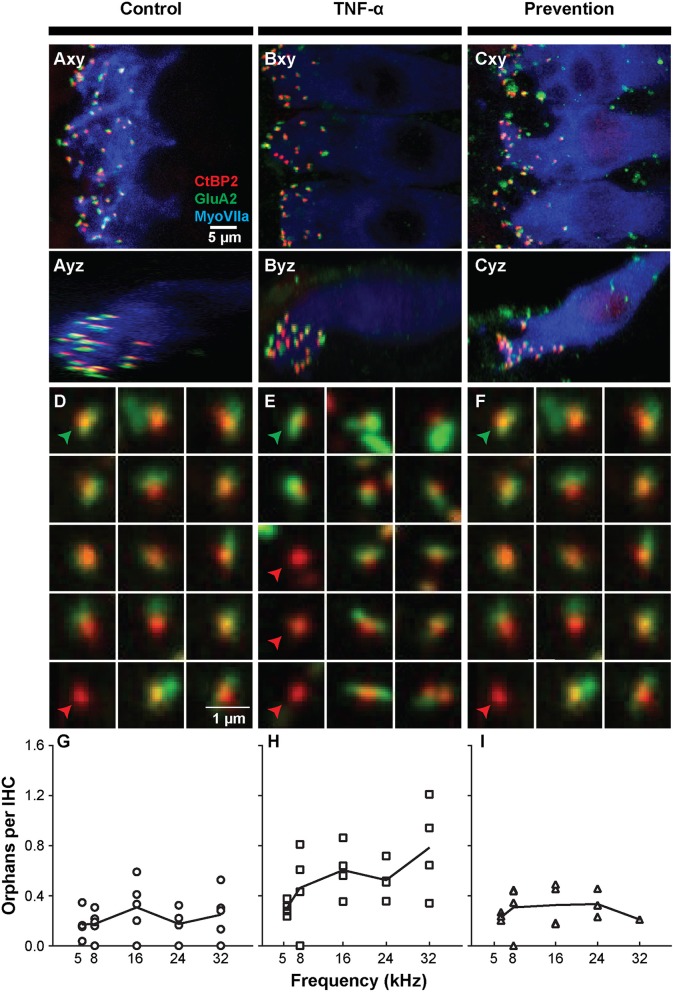
Intracochlear TNF-α perfusion increases the number of orphaned synapses at the basal pole of inner hair cells. Left column shows results from the control group (perfused with artificial perilymph); middle column shows results from the TNF-α group; right column shows results from the prevention group (animals received etanercept 2 h prior to receiving TNF-α). **(A–C)** Representative confocal images of inner hair cells (IHCs) immunostained for myosin VIIa (blue) with pre-synaptic ribbons stained for CtBP2 (red) and post-synaptic terminals stained for GluA2 (green). Images from the 32-kHz region show maximum projections from “surface” views (*xy*) of three adjacent IHCs and side views (*yz*) of the same confocal image stacks. Green arrowheads point to a paired synapse, i.e., CtBP2- and GluA2-positive puncta, indicating presence of a communicating post-synaptic terminal. Red arrowheads point to unpaired (orphaned) synapses, i.e., CtBP2-positive puncta. Scale bar in **(A)** also applies to **(B,C)**. **(D–F)** Custom software automatically detects and segments ribbon synapses based on presence of the CtBP2 label and associated fluorescence signal. Representative “thumbnail” images depicting detected ribbons in control **(D)**, TNF-α **(E)**, and prevention **(F)** conditions demonstrate the reduced number of ribbons with paired post-synaptic terminals in the TNF-α condition relative to the control and prevention conditions. Each thumbnail depicts the x-y projection of the voxel space within 1 μm of a CtBP2-indicative punctum. Green arrowhead, paired synapses; red arrowhead, unpaired (orphaned) synapse. **(G–I)** The number of orphaned ribbon synapses per inner hair cell at 5.6, 8, 16, 24, 32 kHz in control (**G**; *n* = 5), TNF-α (**H**; *n* = 4), and prevention (**I**; *n* = 4) groups. Each symbol in a panel represents an individual animal. There is a significant difference between the three experimental groups at 24 kHz [χ^2^ (2) = 9.05, *p* = 0.001] and 32 kHz [χ^2^ (2) = 7.06, *p* = 0.018]. Further, the number of orphaned synapses at 32 kHz is statistically greater in the TNF-α group relative to the control group [χ^2^ (1) = 4.86, *p* = 0.03] and prevention group [χ^2^ (1) = 5.33, *p* = 0.03]. Similarly, the number of orphaned synapses at 24 kHz is statistically greater in the TNF-α group relative to the control group [χ^2^ (1) = 6.0, *p* = 0.02].

The observed functional effects of TNF-α and etanercept were corroborated by findings from morphologic assessment of inner hair cell synapses (Figure 4). The active region of a healthy inner hair cell afferent synapse is characterized by the presence of both (1) an electron-dense pre-synaptic ribbon, and (2) a post-synaptic AMPA glutamate receptor, located at the proximal end of the spiral ganglion auditory nerve fiber that receives an auditory signal from the hair cell and sends it to the brain (Figures 4A–C). In our study, ribbon synapses were immunolabeled with an antibody to CtBP2, an important synaptic ribbon protein, and post-synaptic receptors were labeled with an antibody to GluA2, an AMPA receptor protein. The respective signals are shown in red and green in Control (Figure 4A), TNF-α (Figure 4B), and Prevention (Figure 4C) groups where Myosin VIIa-expressing inner hair cells are labeled in blue. Custom software (29) automatically detected and segmented CtBP2-expressing ribbons into separate squares, each depicting the x-y-projection of a 1 μm^3^ voxel space centered at a detected ribbon (Figures 4D–F). Such segmentation analyses and subsequent counting of projections including CtBP2 signal but not GluA2 signal (i.e., unpaired ribbon synapses, or “orphaned” synapses) was performed at 5.6, 8, 16, 24, and 32 kHz regions (Figures 4G–I) and revealed a statistically significant difference in the number of orphaned ribbons per inner hair cell between the three experimental groups at 24 kHz [χ^2^ (2) = 9.05, *p* = 0.001] and 32 kHz [χ^2^ (2) = 7.06, *p* = 0.018] but not at 5.6 kHz [χ^2^ (2) = 5.01, *p* = 0.07], 8 kHz [χ^2^ (2) = 2.83, *p* = 0.26], or 16 kHz [χ^2^ (2) = 4.02, *p* = 0.14]. Further analysis at 32 kHz revealed a statistically significant increase in the number of orphans in the TNF-α group compared to the control group [χ^2^ (1) = 4.86, *p* = 0.03] and prevention group [χ^2^ (1) = 5.33, *p* = 0.03]; importantly, there was no difference between the control and prevention groups [χ^2^ (1) = 0.24, *p* = 0.73]. Similarly, at 24 kHz, there was a statistically significant difference in the number of orphans between the TNF-α and control groups [χ^2^ (1) = 6.0, *p* = 0.02] and a similar trend between the TNF-α and prevention groups [χ^2^ (1) = 4.08, *p* = 0.05], but not between the control and prevention groups [χ^2^ (1) = 3.84, *p* = 0.06]. No significant differences were observed in number of inner or outer hair cells or in the overall number of ribbons across groups. This finding is consistent with our physiologic result, and the previously published results that TNF-α preferentially damages the basal turn of the cochlea (38, 39), supporting the hypotheses that TNF-α has the potential to exert a toxic effect on inner ear structure when present at high concentrations in the inner ear, and that etanercept may protect against the associated degenerative effects if administered in a timely manner.

## Discussion

Here we investigate the structural and functional effects of *in vivo* intracochlear perfusion of TNF-α through the cochlea in guinea pigs to further elucidate the role of TNF-α in SNHL. Our results show that acute elevations in intracochlear levels of TNF-α cause a reduction in the CAP amplitude at 32 kHz in addition to synaptic degeneration along the cochlear length within 6 h of TNF-α administration, without significantly affecting audiometric thresholds. Importantly, these effects were abrogated when etanercept, a TNF-α-blocker, was administered systemically prior to TNF-α perfusion. These findings support the hypothesis that VS-induced SNHL may result in part from TNF-α-containing tumor secretions passing into the cochlea via the internal auditory canal.

The hypothesis that TNF-α may play a role in VS-induced SNHL comes from several studies showing that (a) VS tumor secretions contain high levels of TNF-α (20), (b) VS tumor size and proximity to the inner ear are poorly correlated with SNHL in VS patients (3), and (c) upregulation of TNF-α in the cochlea in response to a variety of etiologies of inner ear disease causes hearing loss (21–25, 42–46). Previous studies have shown upregulated levels of TNF-α following exposure to high levels of noise, implicating TNF-α in acoustic trauma (24, 25, 46). In addition, increased expression of TNF-α has been observed in the modiolus, spiral ganglion, and stria vascularis in mouse models of presbycusis (22), and other groups have shown that vibration-induced SNHL results in upregulation of intracochlear levels of TNF-α (21). Direct inoculation of cerebrospinal fluid with Streptococcus pneumoniae in Mongolian gerbil model led to an increase in TNF-α circulation that was directly associated with bacterial meningitis and elevations in auditory brainstem response (ABR) thresholds (23). Activation of TNF-α is also observed in rodents treated with cisplatin (42, 43) and gentamicin (44, 45), both of which have known structural and functional ototoxic effects. Our findings contribute to this body of work by suggesting that the acute effects of TNF-α exposure on cochlear structure and function may be specific to synaptic degeneration and reduction in CAP amplitudes, respectively. Future work should aim to systematically document the effects of TNF-α exposure on intracochlear structure and function in all of the aforementioned etiologies of SNHL to reveal a potential pattern across diseases, and investigate the longer-term otologic effects of TNF-α upregulation in the inner ear.

The present *in vivo* experiments follow up on our previous *ex vivo* investigations into the effects of applying both recombinant TNF-α and human VS secretions to murine cochlear explants (20). In the *ex vivo* studies, while secretions from specific VS tumors caused inner hair cell damage, outer hair cell damage, and nerve fiber disorganization, application of recombinant TNF-α caused nerve fiber disorganization and a decrease in neurite counts without evidence of any hair cell damage. The present *in vivo* studies were also conducted using recombinant TNF-α, and the observed synaptic degeneration corroborates the findings of nerve fiber disorganization in the previous *ex vivo* studies. Thus, it is likely that the VS secretion-induced hair cell damage observed in our previous *ex vivo* studies occurred due to a longer or different method of exposure, and/or was mediated by a different ototoxic secreted factor or some other mechanism altogether. Relevantly, it is known from studies conducted in the central nervous system that overexpression of TNF-α can cause acute synaptic degeneration via glutamate-mediated cytotoxicity. Specifically, excess TNF-α production has been shown to lead to neuronal death (47) mediated predominantly by TNF receptor 1 activation (48), and it has been demonstrated that TNF-α's neurotoxic action most likely targets the glutamatergic system, potentiating AMPA-induced excitotoxicity (49). In particular, Cueva Vargas et al. (50) reported that TNF-α-induced retinal ganglion cell death in glaucoma may be specifically mediated by increased expression of Ca^2+^-permeable AMPA receptors that is caused by selective downregulation of the GluA2 subunit of the AMPA receptors in these neurons. Returning to the peripheral auditory system, it is widely accepted that glutamate is the afferent neurotransmitter responsible for communication between inner hair cells and spiral ganglion neuron terminals in the cochlea (51) and that AMPA receptors are abundant and critically important for fast synaptic transmission in the guinea pig cochlea (52). Because we specifically immunostained for presence of the GluA2 subunit of AMPA receptors, our observation of a reduced number of paired synapses in the TNF-α group relative to the artificial perilymph and prevention experimental groups is consistent with Cueva Vargas et al.'s finding in retinal ganglion cells. Importantly, it is likely that we would have observed more widespread degeneration if we had performed longitudinal experiments to study the long-term effects of chronic overexposure of the inner ear to TNF-α, as other reports (including those from our own lab as described above) have demonstrated deleterious effects on hair cells as well. Future studies should further investigate the mechanisms underlying both hair cell and neuronal damage observed in response to VS secretion exposure, and should be designed to distinguish between acute and chronic effects of the presence of VS secretions in the inner ear.

In a previous *in vivo* study on TNF-α-induced intracochlear cytotoxicity, Keithley et al. (53) investigated whether the mechanism underlying TNF-α-induced SNHL may involve TNF-α-induced recruitment of circulatory leukocytes to the inner ear, as it is known that the inner ear's rapid inflammatory response to antigen/pathogen invasion can cause hearing loss and irreversible damage to the inner ear's mechanosensory structures (54, 55). While they did find that perfusion of high concentrations (2 μg/mL administered over the course of 4 days) of TNF-α induced recruitment of inflammatory cells to the perivascular space around the spiral modiolar vein, they did not observe significant differences between the TNF-α and vehicle experimental groups in click-evoked ABR thresholds *in vivo*, and they observed only minimal differences between these groups in inner and outer hair cell counts *in vitro*. These findings are consistent with our present results; specifically, we also did not observe significant differences in CAP thresholds between the TNF-α and control groups, and did not observe significant differences between these groups in hair cell counts. Keithley et al. did not comment on CAP amplitudes or synaptic integrity in their report. While we cannot directly compare Keithley et al.'s results with ours, because we measured tone-evoked CAPs, both studies nonetheless have important parallels, and reach similar conclusions. Taken together, our current *in vivo* results and previously published *ex vivo* results on application of human VS secretions to mouse cochlear explants suggest that in cases of VS-induced SNHL where inner and outer hair cell death are observed, the hair cell degeneration cannot be explained by acute TNF-α-mediated cytotoxicity alone.

Our finding that administration of etanercept, a TNF-α-blocker, protected the inner ear from TNF-α induced structural and functional transformation is consistent with a large body of literature showing that blocking TNF-α in several contexts of SNHL can prevent subsequent SNHL (56–59). One hypothesis regarding the mechanism of action of TNF-α is that it induces a pre-constrictive state throughout the cochlea's microcirculatory system, thereby decreasing cochlear blood flow and inducing cochlear ischemia. Etanercept has been shown to protect against this vasoconstriction by increasing cochlear blood flow when administered both pre- and post-trauma-inducing stimulus exposure, suggesting its utility for both protection against and rescue from the harmful effects of TNF-α exposure. Ihler et al. (60) demonstrated etanercept's protective effect against TNF-α mediated reductions in cochlear blood flow, while Arpornchayanon et al. (56) showed that systemic administration of etanercept following high noise exposure increased strial capillary blood flow and preserved ABR thresholds relative to animals that were exposed to noise without subsequent etanercept treatment. Importantly, etanercept has already been used pre-clinically for treating classic inflammatory types of SNHL such as labyrinthitis (59). However, a small pilot clinical study did not find etanercept effective for treating autoimmune inner ear disease (57). Our findings are consistent with those showing etanercept's protective effect—we found that providing etanercept prior to TNF-α perfusion promoted preservation of CAP amplitudes and synaptic integrity. Indeed, the animals that received etanercept were not significantly different from the artificial perilymph-receiving controls in their CAP amplitudes and number of orphaned ribbon synapses. Taken together, our results suggest that TNF-α-induced synaptopathy may be prevented by etanercept administration. Although precise mechanisms of this therapeutic effect remain to be determined, it is possible that etanercept prevents TNF-α-induced reduction in cochlear blood flow and local anoxia. These findings motivate further investigation into whether and how etanercept can be used to mediate the effects of trauma-induced TNF-α upregulation in the inner ear to prevent the subsequent irreversible damage.

An important limitation of the present study is the acute nature of the experimental design; specifically, hearing was assessed up to 6 h post-TNF-α perfusion, when animals were immediately sacrificed and ears extracted for histology. By contrast, in previous *in vivo* and *in vitro* studies assessing TNF-α's ototoxic effect on the organ of Corti, animals or cochlear explants, respectively, were kept alive for several days during or after TNF-α treatment before histological processing and assessment (38, 53). The *in vitro* studies showed significant deterioration over the course of several days of incubation in TNF-α-doped culture medium, and significant differences between 2, 4, and 8 days specifically (38). The acute exposure and short experimental timeline in the present studies may explain the observed minimal changes in hearing sensitivity, and inner and outer hair cell integrity.

In summary, although we did not observe any direct effects of TNF-α on CAP or DPOAE thresholds or on hair cell counts, our experiments did reveal that TNF-α specifically caused a decrease in CAP amplitudes accompanied by an increase in the number of unpaired or orphaned ribbon synapses within 6 h of exposure. This finding suggests a pathologic profile that is similar to what is observed in primary cochlear neuropathy—in the latter disease, reductions in ABR Wave I amplitudes and synaptic degeneration are observed with minimal changes in ABR Wave I and DPOAE thresholds and minimal loss of inner and outer hair cells in mice (34, 40) and guinea pigs (29). These synaptic changes occur rapidly, within hours of noise exposure. Moreover, age-related synaptic degeneration long precedes age-related changes in audiometric thresholds or hair cell counts in mice (41). Importantly, both cochlear neuropathy patients and VS patients typically experience difficulties with word recognition and speech-in-noise tasks, which are thought to reflect loss of peripheral axons (37). Future work should investigate the effects of *in vivo* chronic intracochlear exposure to TNF-α and assessment of hearing and cochlear morphology at several sequential time points, in addition to further exploring etanercept's ability to protect against TNF-α-induced SNHL, and rescue hearing function post-exposure to TNF-α. Given that the brainstem is also located near the cerebellopontine angle, future *in vitro* and *in vivo* brain studies should investigate the brainstem's potential vulnerability to high levels of TNF-α in VS secretions.

## Data Availability Statement

The datasets generated for this study are available on request to the corresponding author.

## Ethics Statement

The animal study was reviewed and approved by Massachusetts Eye and Ear Institutional Animal Care and Use Committee.

## Author Contributions

KS conceived of the project and supervised all the work. KS and MS designed experiments. MS and SK performed electrophysiological measurements. SK and RL performed confocal immunohistochemistry and synaptic counting. SK, RL, and LL performed cochlear microdissections. SK, JI, MS, RL, and KS analyzed data. JI and KS wrote the manuscript. All authors edited the manuscript and approved the final version.

### Conflict of Interest

The authors declare that the research was conducted in the absence of any commercial or financial relationships that could be construed as a potential conflict of interest.

## Supplementary Material

Supplemental Figure 1 Mean DP amplitude (2f1-f2) vs. level functions for f2 = 16 kHz **(A)**, 24 kHz **(B)** and 32 kHz **(C)** at 6 h post-perfusion for the three groups. The control group was perfused with artificial perilymph. The prevention group received etanercept 2 h prior to intracochlear TNF-α perfusion. The differences among groups were not statistically significant. Error bars represent SEM. Figure legend in **(A)** also applies to **(B,C)**.

## Abbreviations

μL: microliter
μV: microvolts
ABR: auditory brainstem response
AMPA: α-amino-3-hydroxy-5-methyl-4-isoxazolepropionic acid
CAP: compound action potential
CtBP2: C-terminal-binding protein 2
dB: decibel
DPOAE: distortion product otoacoustic emission
EDTA: ethylenediaminetetraacetic acid
GluA2: glutamate ionotropic receptor AMPA type subunit 2
H&E: hematoxylin and eosin
h: hours
kg: kilogram
kHz: kilohertz
mg: milligram
mL: milliliter
mm: millimeter
mM: millimolar
ms: millisecond
NHS: normal horse serum
PBS: phosphate buffered saline
PFA: paraformaldehyde
SNHL: sensorineural hearing loss
SPL: sound pressure level
TNF-α: tumor necrosis factor-alpha
VS: vestibular schwannoma.
