# Supplementary figures and images for: Intracochlear Perfusion of Tumor Necrosis Factor-Alpha Induces Sensorineural Hearing Loss and Synaptic Degeneration in Guinea Pigs
Source: Front Neurol. 2020 Feb 10;10:1353. doi: 10.3389/fneur.2019.01353 (PMC7025643; doi:10.3389/fneur.2019.01353)

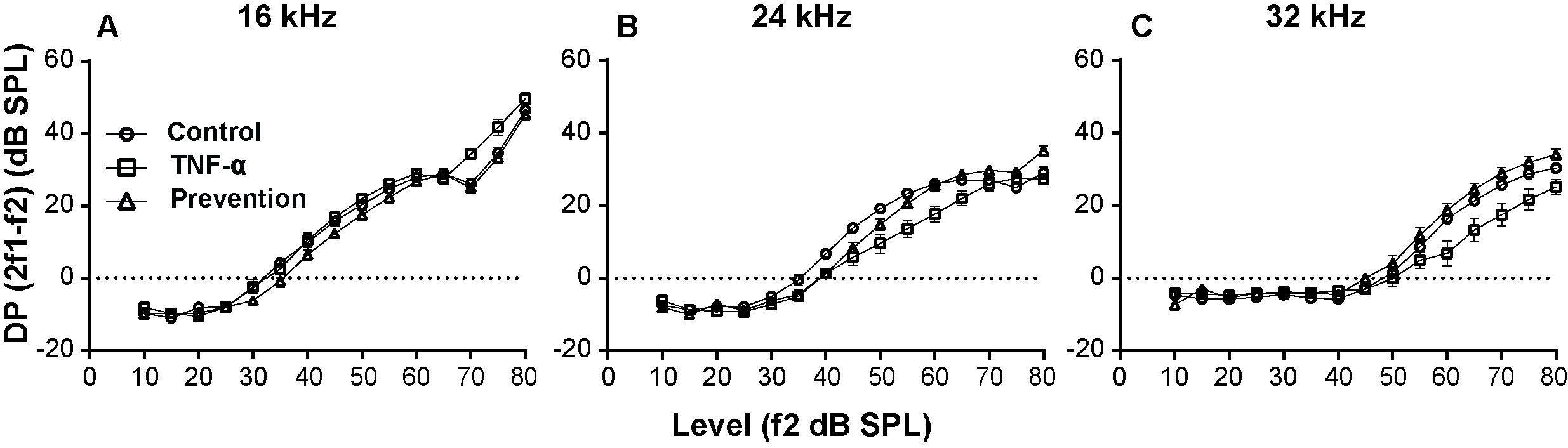

Supplement: Supplemental Figure 1 — Mean DP amplitude (2f1-f2) vs. level functions for f2 = 16 kHz (A), 24 kHz (B) and 32 kHz (C) at 6 h post-perfusion for the three groups. The control group was perfused with artificial perilymph. The prevention group received etanercept 2 h prior to intracochlear TNF-α perfusion. The differences among groups were not statistically significant. Error bars represent SEM. Figure legend in (A) also applies to (B,C). [file Image_1.tif]
